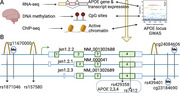# Identification of a specific *APOE* transcript and functional elements associated with Alzheimer's disease

**DOI:** 10.1002/alz70855_099140

**Published:** 2025-12-23

**Authors:** Liang Ma

**Affiliations:** ^1^ University of Texas Health Science Center at San Antonio, San Antonio, TX, USA

## Abstract

**Background:**

The *APOE* gene is the strongest genetic risk factor for late‐onset Alzheimer's Disease (LOAD). However, the gene regulatory mechanisms at this locus remain incompletely characterized.

**Methods:**

To identify novel AD‐linked functional elements within the *APOE* locus, we integrated SNP variants with multi‐omics data from human postmortem brains including 2,179 RNA‐seq samples from 3 brain regions and two ancestries (European and African), 667 DNA methylation samples, and ChIP‐seq samples. Additionally, we plotted the expression trajectory of *APOE* transcripts in human brains during development.

**Results:**

We identified an AD‐linked *APOE* transcript (jxn1.2.2) particularly observed in the dorsolateral prefrontal cortex (DLPFC). The *APOE* jxn1.2.2 transcript is associated with brain neuropathological features, cognitive impairment, and the presence of the *APOE4* allele in DLPFC. We prioritized two independent functional SNPs (rs157580 and rs439401) significantly associated with jxn1.2.2 transcript abundance and DNA methylation levels. These SNPs are located within active chromatin regions and affect brain‐related transcription factor‐binding affinities. The two SNPs shared effects on the jxn1.2.2 transcript between European and African ethnic groups.

**Conclusion:**

The novel *APOE* functional elements provide potential therapeutic targets with mechanistic insight into the disease etiology.